# REDD1 Activates a ROS-Generating Feedback Loop in the Retina of Diabetic Mice

**DOI:** 10.1167/iovs.19-26606

**Published:** 2019-05

**Authors:** William P. Miller, Allyson L. Toro, Alistair J. Barber, Michael D. Dennis

**Affiliations:** 1 Department of Cellular and Molecular Physiology, Penn State College of Medicine, Hershey, Pennsylvania, United States; 2 Department of Ophthalmology, Penn State College of Medicine, Hershey, Pennsylvania, United States

**Keywords:** antioxidants, diabetes, oxidative stress, retina, vision

## Abstract

**Purpose:**

The present study was designed to evaluate the role of the stress response protein REDD1 in diabetes-induced oxidative stress and retinal pathology.

**Methods:**

Wild-type and REDD1-deficient mice were administered streptozotocin to induce diabetes. Some mice received the antioxidant *N*-acetyl-l-cysteine (NAC). Visual function was assessed by virtual optometry. Retinas were analyzed by Western blotting. Reactive oxygen species (ROS) were assessed by 2,7-dichlorofluoroscein. Similar analyses were performed on R28 retinal cells in culture exposed to hyperglycemic conditions, NAC, and/or the exogenous ROS source hydrogen peroxide.

**Results:**

In the retina of diabetic mice, REDD1 expression and ROS were increased. In cells in culture, hyperglycemic conditions enhanced REDD1 expression, ROS levels, and the mitochondrial membrane potential. However, similar effects were not observed in the retina of diabetic mice or cells lacking REDD1. In the retina of diabetic mice and cells exposed to hyperglycemic conditions, NAC normalized ROS and prevented an increase in REDD1 expression. Diabetic mice receiving NAC also exhibited improved contrast sensitivity as compared to diabetic controls. Hydrogen peroxide addition to culture medium increased REDD1 expression and attenuated Akt/GSK3 phosphorylation in a REDD1-dependent manner. In REDD1-deficient cells exposed to hyperglycemic conditions, expression of a dominant negative Akt or constitutively active GSK3 increased the mitochondrial membrane potential and promoted ROS.

**Conclusions:**

The findings provide new insight into the mechanism whereby diabetes-induced hyperglycemia causes oxidative stress and visual dysfunction. Specifically, hyperglycemia-induced REDD1 activates a ROS-generating feedback loop that includes Akt/GSK3. Thus, therapeutic approaches targeting REDD1 expression and ROS may be beneficial for preventing diabetes-induced visual dysfunction.

Hyperglycemia is a major causative factor in the development of complications associated with diabetes.^1^ The Diabetes Control and Complications Trial demonstrated that intensive glycemic control is associated with a reduction in both the onset and progression of diabetic retinopathy (DR),^2^ yet the molecular events that contribute to early diabetes-induced retinal dysfunction remain incompletely understood. A unifying theory for the pathophysiology of diabetic complications suggests that the principle pathways responsible for hyperglycemia-induced tissue damage are all linked to the overproduction of reactive oxygen species (ROS).^3^ Oxidative stress results from an imbalance between the production of ROS, such as superoxide anion (O_2_^−^), hydroxyl radical (OH), hydrogen peroxide (H_2_O_2_), and singlet oxygen (^1^O_2_), and the antioxidant defense system. In diabetes, an increase in the production of ROS and an impaired capacity to reduce free radicals contributes to retinal pathogenesis.^4^ Experimental evidence demonstrates that hyperglycemia-induced mitochondrial superoxide production drives the development of oxidative stress in the retina.^5^,^6^ In fact, overexpression of the mitochondrial superoxide dismutase is sufficient to prevent diabetes-induced oxidative stress and the development of acellular capillaries in the retina.^6^,^7^

Hyperglycemic conditions activate a multicomponent feedback loop that promotes mitochondrial ROS production.^8^ Hyperglycemia provides an abundance of substrate for glucose oxidation, leading to increased generation of NADH and pyruvate, enhanced electron transport chain flux, and a high inner mitochondrial trans-membrane potential (ΔΨ_m_).^9^,^10^ Mitochondrial ROS generation is dependent on a high proton gradient, as O_2_^−^ and H_2_O_2_ formation dramatically increase when ΔΨ_m_ exceeds a threshold level.^11^ Mitochondrial hexokinase (HK) activity is an important regulator of ΔΨ_m_. Specifically, the enzyme provides local ADP recycling through the VDAC (voltage-dependent anion channel)/ANT (adenine nucleotide translocator) complex to the F_1_F_0_ ATP synthase complex, which phosphorylates the ADP at the expense of ΔΨ_m._^12^ The association of HK with VDAC is reduced by glycogen synthase kinase 3β (GSK3β)-dependent phosphorylation of VDAC.^13^ In turn, GSK3β activity is inhibited by Akt-dependent phosphorylation of the GSK3 N-terminus, which reduces kinase activity by obstructing substrate binding.^14^,^15^ In response to hyperglycemic conditions, protein phosphatase 2A (PP2A) inhibits Akt kinase activity by dephosphorylation of the kinase.^8^ This reduction in Akt activity leads to increased GSK3β-dependent phosphorylation of VDAC, reduced mitochondrial association with HK, and increased ROS production.

The stress response protein regulated in development and DNA damage 1 (REDD1) promotes association of PP2A with Akt, leading to site-specific dephosphorylation of the kinase and subsequent reduction in Akt-mediated phosphorylation of GSK3.^16^ Thus, we speculated that REDD1 expression may be a critical element in the feedback loop that increases ROS production in response to hyperglycemic conditions. Since its discovery, REDD1 has been linked to the regulation of ROS.^17^–^20^ Our laboratory recently demonstrated that REDD1 expression is increased in the retina of diabetic mice by hyperglycemia,^21^ coincident with attenuated Akt kinase activity and increased retinal cell death.^22^ In the retina of diabetic mice lacking REDD1, Akt activity remains elevated and cell death is similar to that observed in nondiabetic controls.^22^ In fact, genetic ablation of REDD1 prevents electroretinogram defects or visual threshold deficits in diabetic mice.^22^ Similarly, REDD1 has also been linked to the development of ischemic proliferative retinopathy.^23^ Remarkably, the DEGAS study demonstrates that intravitreal injection of a siRNA targeting the REDD1 mRNA is more effective than laser photocoagulation in improving visual acuity in patients with diabetic macular edema.^24^ Improved vision in patients treated with a siRNA targeting the REDD1 mRNA occurred in the absence of altered anatomical features or fluorescein leakage, suggesting a mechanism independent of changes in vascular permeability. In the present study, we investigated the mechanism responsible for the protective effects of REDD1 deletion on diabetes-induced visual dysfunction.

## Methods

### Animals

Male C57BL6/N mice (Charles River, Wilmington, MA, USA) and wild-type/REDD1 knockout B6;129 mice^23^ were administered either 50 mg/kg streptozotocin (STZ) or sodium citrate buffer for 5 consecutive days to induce diabetes. Diabetic phenotype was assessed with fasting blood glucose levels >250 mg/dL. Approximately 2 g/kg per day *N*-acetyl-l-cysteine (NAC) was administered via drinking water. All procedures were approved by the Penn State College of Medicine Institutional Animal Care and Use Committee.

### Cell Culture

CRISPR/Cas9 genome editing to generate a stable R28 cell line deficient in REDD1 was previously described.^22^ REDD1 knockout mouse embryonic fibroblasts (MEF) were provided by L. Ellisen (Harvard Medical School, Boston, MA, USA). Cells were cultured on plates (CellBIND; Corning, Corning, NY, USA) with Dulbecco's modified Eagle's medium (DMEM) containing 5 mM glucose and 10% fetal bovine serum (FBS). R28 medium was supplemented with 1× MEM nonessential amino acids, 1× MEM vitamin solution, and 0.14% gentamycin. MEF medium was supplemented with 1% penicillin/streptomycin. For studies on the effects of hyperglycemic conditions, cell culture medium contained either 30 mM glucose or 5 mM glucose with 25 mM mannitol as an osmotic control. Where indicated, cells were cultured in medium without FBS for 24 hours. In specific studies, culture medium was supplemented with 100 nM insulin (Lilly, Indianapolis, IN, USA), 10 mM NAC (Sigma-Aldrich Corp., St. Louis, MO, USA), or 1 mM hydrogen peroxide. Cells were transfected with pCMV5 vector, pCMV-HA-REDD1 (human), pCMV-HA-GSK3-S9A (no. 14754; Addgene, Watertown, MA, USA) or pCMV-HA-AKT-K147M (no. 16243; Addgene) plasmids using a reagent (Lipofectamine 2000; Life Technologies, Carlsbad, CA, USA). To evaluate mitochondrial membrane potential, cells were transferred to Opti-MEM reduced-serum media lacking phenol red and stained with 1 μg/mL dye (JC-1; Life Technologies).

### ROS Measurement

Eyes were harvested, embedded in OCT, and flash frozen. Cryosections (10 μm) were fixed with 2% paraformaldehyde in PBS (pH 7.4). Cryosections were stained with 1.6 μM Hoechst and 10 μM 2,7-dichlorofluoroscein (DCF). Sections were imaged using a confocal laser microscope (λex = 504 nm) (Leica SP8; Leica, Wetzlar, Germany). Alternatively, retinas were also homogenized in lysis buffer as previously described.^22^ Lysates were centrifuged at 1500*g* for 3 minutes, and the supernatant was exposed to 10 μM DCF. Fluorescence was measured using a plate reader (Spectra Max M5; Molecular Devices, San Jose, CA, USA) (ex/em = 504/529 nm). ROS were assessed in cells in culture using a DCF diacetate (DCFDA) assay kit (DCFDA Cellular ROS Detection Assay kit; Abcam, Cambridge, United Kingdom). To assess mitochondrial superoxide, cells were cultured on 35 mm poly-d-lysine–coated glass-bottom culture dishes with No. 1.5 coverslip (MatTek Corp., Ashland, MA, USA) and exposed to 5 μM MitoSOX (Thermo Fisher Scientific, Waltham, MA, USA) prepared in Hanks' buffered salt solution supplemented with 1.3 mM calcium and 0.9 mM magnesium.

### Cell Death ELISA

Cells or retinas were homogenized in lysis buffer and relative cell death was evaluated using an ELISA kit (Cell Death Detection ELISA Kit; Roche, Basel, Switzerland) as previously described.^22^

### Western Blot Analysis

Retinas were flash frozen in liquid nitrogen and homogenized in 250 μL of lysis buffer as previously described.^22^ Cell lysates and retinal homogenates were fractionated using 4%–20% gels (Criterion Precast; Bio-Rad Laboratories, Hercules, CA, USA). Proteins were transferred to a polyvinylidene fluoride membrane, reversibly stained to assess protein loading (Pierce, Waltham, MA, USA), blocked in 5% milk in Tris-buffered saline Tween 20, and evaluated with the appropriate antibodies (Table).

**Table:** Antibodies Used for Western Blot Analysis

Antibodies*	**Cat No.**	**Lot No.**	**Dilution**
Cell Signaling Technology			
Cleaved caspase 3	9664S	21	1 to 1000
AKT-p (T308)	9275L	5	1 to 1000
AKT-T	9272S	27	1 to 1000
GSK3 α/β-p (S21/9)	8566P	3	1 to 1000
GSK3 β-T	12456S	3	1 to 1000
Proteintech			
REDD1-T	10638-1-AP	51272	1 to 500
Santa Cruz Biotechnology			
GAPDH	sc-32233	K0315	1 to 5000
α-tubulin	sc-32293	C0112	1 to 5000
HA-(Y11)	sc-805	I2315	1 to 500
Bethyl Laboratories			
Goat anti-rabbit IgG-heavy and light	A120-101P	40	1 to 10000
Goat anti-mouse IgG-heavy and light	A90-116P	39	1 to 10000

* Supplier locations: Cell Signaling Technology (Danvers, MA, USA), Proteintech Group, Inc. (Rosemont, IL, USA), Santa Cruz Biotechnology, Inc. (Dallas, TX, USA), and Bethyl Laboratories, Inc. (Montgomery, TX, USA).

### RNA Isolation and Quantitative PCR (qPCR)

Total RNA was extracted with TRIzol (Invitrogen, Carlsbad, CA, USA). RNA (1 μg) was reverse transcribed using a reverse transcription kit (High Capacity cDNA Reverse Transcription Kit; Applied Biosystems, Foster City, CA, USA) and subjected to quantitative real-time PCR (Q12K Flex Real-Time PCR System; Applied Biosystems) using a PCR kit (QuantiTect SYBR Green Master Mix; Qiagen, Hilden, Germany) as previously described.^25^ Primers for REDD1 were as follows: 5′-TTCGAGAGGCAGATCGCT-3′ and 5′-GAAGAGGAGGACGAGAAACGA-3′. Mean cycle threshold (C_T_) values for REDD1 and GAPDH were determined for control and experimental samples. Changes in REDD1 mRNA expression were normalized to GAPDH mRNA expression using the 2^−ΔΔCT^ calculation as previously described.^26^

### Behavioral Assessment of Visual Thresholds

A virtual optomotor system (OptoMotry; Cerebral Mechanics, Irvine, CA, USA) was used to assess visual function in mice as previously described.^27^ Spatial frequency (SF) and contrast sensitivity (CS) thresholds were identified as the highest values that elicited a reflexive head movement. CS was evaluated at an SF of 0.092 cycles/deg. SF was assessed at 100% contrast. CS was expressed as an inverse percentage to make data interpretation more intuitive.

### Statistical Analysis

Data are expressed as mean ± SEM. Data were analyzed overall with ANOVA, and trend test and pairwise comparisons were conducted with the Tukey test for multiple comparisons. A *t*-test was used to compare mean differences between 5 and 30 mM glucose concentrations (see Fig. 3). Significance was defined as *P* < 0.05 for all analyses.

## Results

### NAC Supplementation Attenuates Retinal ROS and REDD1 Expression in a Model of Type 1 Diabetes

**Figure 1:**
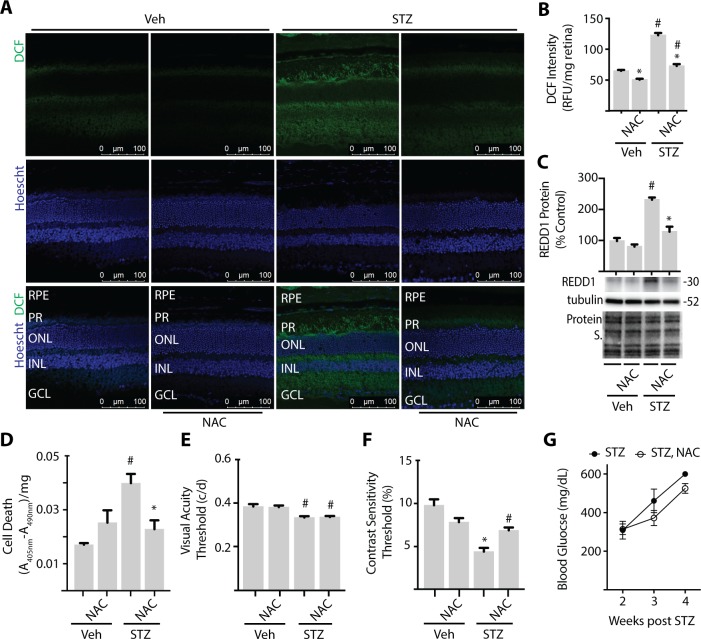
NAC supplementation prevents retinal cell death and improves contrast sensitivity in diabetic mice. C57BL/6N mice were administered STZ or vehicle (Veh) for 5 consecutive days. Some mice received approximately 2 g/kg NAC via drinking water. Analysis was performed after 4 weeks of diabetes with or without NAC supplementation. (A) Whole-eye sagittal cross sections were exposed to 10 μmol/L DCF. (B) DCF fluorescence was quantified in retinal lysates. (C) REDD1 and α-tubulin expression were evaluated in retinal lysates by Western blotting. Gel loading was assessed via protein stain. Protein molecular mass (kDa) is indicated at right of blots. (D) Relative cell death was assessed by ELISA for cytoplasmic nucleosomes. Visual function was assessed by virtual optometry. Visual acuity (E) and contrast sensitivity (F) thresholds were obtained on consecutive days. The contrast sensitivity threshold is expressed as a reciprocal value. (G) Blood glucose was evaluated 2 to 4 weeks after mice were administered STZ. Results are from three independent experiments. Within each experiment three to four independent samples were analyzed. Values are means + SEM. Statistically significant differences (P < 0.05) are denoted by * for Veh versus NAC and # for Veh versus STZ. c/d, cycles/degree; RFU, relative fluorescent units; A, absorbance; RPE, retinal pigment epithelium; PR, photoreceptor; ONL, outer nuclear layer; INL, inner nuclear layer; GCL, ganglion cell layer.

To evaluate the role of diabetes-induced oxidative stress in retinal cell death and visual dysfunction, STZ-diabetic mice were administered NAC over a 4-week period of diabetes. NAC is an antioxidant and glutathione precursor, and supplementation has been previously shown to have beneficial effects on DR pathology.^28^,^29^ To evaluate oxidative stress, retinal cryosections were exposed to the fluorescent ROS indicator DCF. Consistent with the previous report,^30^ diabetes increased retinal ROS as assessed by DCF fluorescence (Fig. 1A, 1B). Quantitative assessment of ROS in retinal tissue lysates demonstrated that NAC reduced ROS levels in both diabetic and nondiabetic mice as compared to vehicle controls (Fig. 1B). In fact, DCF fluorescence was similar in the retina of diabetic mice treated with NAC and nondiabetic controls. Consistent with our previous studies,^21^,^22^ REDD1 protein expression (Fig. 1C) and cell death (Fig. 1D) were also increased in the retina of diabetic mice. However, NAC supplementation prevented diabetes-induced retinal REDD1 protein expression and cell death (Fig. 1C–1D). TUNEL-positive nuclei and cells containing positive immunoreactivity for the active form of caspase 3 have been observed in the retina of diabetic rodents during the first 4 weeks of hyperglycemia.^31^,^32^ The position of cells containing activated caspase 3 was consistent with bipolar neurons, amacrine cells, and retinal ganglion cells, and their colocalization with the NeuN and tyrosine hydroxylase antigens further supports that these cells were neuronal.^33^

### NAC Supplementation Improves Contrast Sensitivity in a Model of Type 1 Diabetes

To determine if there was a functional effect of NAC supplementation on vision, we performed behavioral optometry. Consistent with our previous report,^22^ visual acuity (Fig. 1E) and contrast sensitivity (Fig. 1F) deficits were observed after 4 weeks of diabetes. NAC supplementation had no effect on visual acuity. However, contrast sensitivity was improved in diabetic mice receiving NAC as compared to diabetic controls, such that there was no difference between diabetic and nondiabetic mice receiving NAC. While there was a trend toward lower blood glucose levels in mice treated with NAC for 4 weeks (*P* = 0.055), blood glucose levels were similar in diabetic mice with and without NAC supplementation (Fig. 1G).

### REDD1 Deletion Is Sufficient to Prevent Diabetes-Induced ROS in Retina

**Figure 2:**
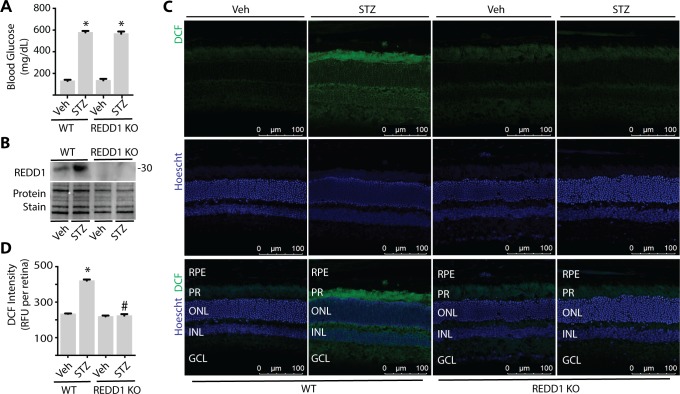
REDD1 deletion is sufficient to prevent diabetes-induced ROS in retina. Diabetes was induced in wild-type (WT) and REDD1 KO B6;129 mice by administration of STZ. All analyses were performed 4 weeks after mice were administered STZ or Veh. (A) Postprandial blood glucose concentrations were evaluated. (B) REDD1 protein expression in retinal lysates was assessed by Western blotting. Protein loading was evaluated by reversible protein stain. Blot shown is representative of three independent experiments. Protein molecular mass (kDa) is indicated at right of blot. (C) Whole eyes were isolated, cryosectioned into sagittally oriented longitudinal cross sections, stained with 1.6 μM Hoechst, exposed to 10 μM DCF, and imaged by confocal laser microscopy. (D) ROS was quantified in supernatants from whole retinal lysates. Results are for three independent experiments. Within each experiment, three to four independent samples were analyzed. Values are means + SEM. *P < 0.05 versus Veh; #P < 0.05 versus WT.

To evaluate the role of REDD1 in diabetes-induced oxidative stress, mice with a germline disruption of REDD1 were administered STZ. After 4 weeks of diabetes, wild-type and REDD1 knockout mice exhibited a similar increase in postprandial blood glucose concentrations (Fig. 2A). REDD1 protein expression was increased in retinal lysates from diabetic wild-type mice, but not detectable in REDD1 knockout mice (Fig. 2B). In the retina of wild-type diabetic mice, DCF fluorescence was increased as compared to nondiabetic controls (Fig. 2C–2D). In nondiabetic REDD1-deficient mice, DCF fluorescence was similar to that observed in nondiabetic wild-type mice. In contrast to the effect of diabetes in wild-type mice, ROS levels were similar in diabetic and nondiabetic REDD1-knockout mice.

**Figure 3:**
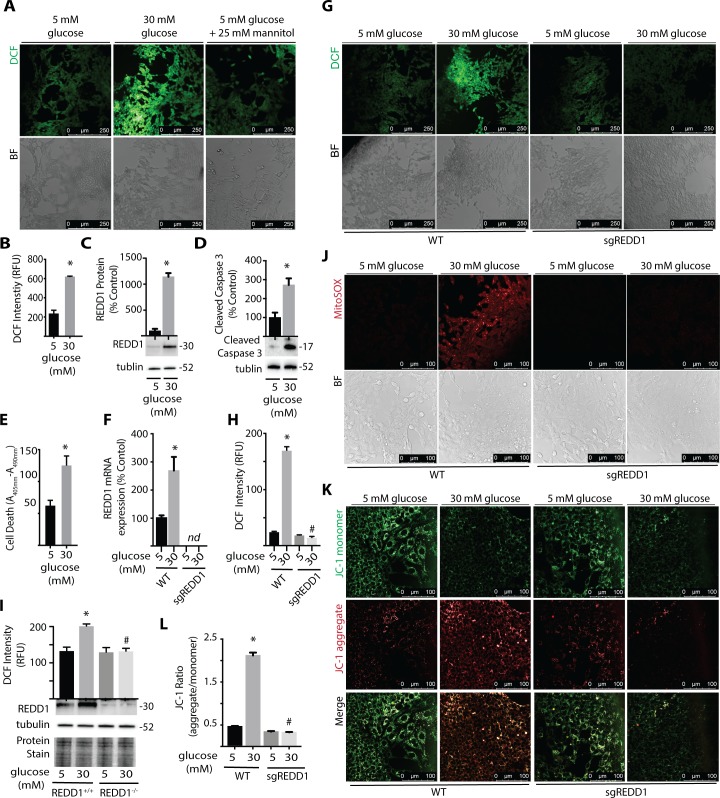
REDD1 deletion is sufficient to prevent hyperglycemia-induced ROS. R28 retinal cells were maintained in DMEM containing 5 mmol/L glucose and supplemented with 10% FBS. (A) Cells were exposed to medium containing 5 mmol/L glucose, 30 mmol/L glucose, or 5 mmol/L glucose plus 25 mmol/L mannitol as an osmotic control for 24 hours. ROS were visualized with DCFDA. DCF fluorescence and bright-field (BF) images are shown. (B) DCF fluorescent intensity per well was quantified using a plate reader. (C, D) REDD1, caspase-3 cleavage, and tubulin protein expression were assessed by Western blotting. Protein molecular mass (kDa) is indicated at the right of blots. (E) Relative cell death was assessed by ELISA of cytoplasmic nucleosomes. R28 WT and REDD1 KO (sgREDD1) cells were maintained in DMEM containing 5 mmol/L glucose supplemented with 10% FBS (F–H, J–L). Cells were exposed to medium containing 5 mmol/L or 30 mmol/L glucose for 24 hours. (F) REDD1 mRNA expression was assessed by qPCR. (G) ROS were visualized with DCFDA, and DCF fluorescent intensity was quantified (H). (I) ROS levels were similarly evaluated in REDD1^+/+^ and REDD1^−/−^ MEF. REDD1 and tubulin protein expression were assessed by Western blotting. Protein loading was evaluated by reversible protein stain. (J) Mitochondrial superoxide was assessed by MitoSOX stain. (K, L) Mitochondrial membrane potential was evaluated with JC-1 dye. JC-1 aggregates and monomers were quantified. Results are representative for three experiments. Within each experiment, 3 to 10 independent samples were analyzed. Values are means + SEM. *P < 0.05 versus Veh; #P < 0.05 versus WT. nd, not detected.

### Hyperglycemic Conditions Increase ROS, REDD1 Expression, and Cell Death in R28 Cells in Culture

To further explore hyperglycemia-induced ROS, R28 retinal cells were maintained in culture medium containing 5 mM glucose and then exposed to medium containing 30 mM glucose. R28 cells are an immortalized rat retinal cell line derived from a mixed population of cells that express both neuronal and glial antigens.^34^ Their generic nature and the ability to culture large numbers of R28 cells make them particularly useful to study the molecular events associated with exposure to hyperglycemic conditions. Cells were stained with DCFDA, which can passively diffuse into cells and become hydrolyzed by cellular esterases to form DCF. In R28 cells exposed to culture medium containing 30 mM glucose, DCF fluorescence was enhanced (Fig. 3A, 3B). In contrast, a similar effect on DCF fluorescence was not observed in cells exposed to culture medium containing 5 mM glucose and 25 mM mannitol as an osmotic control (Fig. 3A). In a previous study, we demonstrated that REDD1 expression was necessary for hyperglycemic conditions to promote cell death.^22^ In support of that study, exposure to culture medium containing 30 mM glucose increased REDD1 protein expression (Fig. 3C) and increased cell death as assessed by caspase 3 cleavage (Fig. 3D) and DNA fragmentation (Fig. 3E).

### REDD1 Deletion Attenuates Glucose-Induced ROS in R28 Cells in Culture

To evaluate the role of REDD1 in hyperglycemia-induced ROS production, wild-type and REDD1-deficient R28 cells were exposed to hyperglycemic conditions. REDD1 mRNA abundance was increased in wild-type cells exposed to hyperglycemic conditions but was not detected upon genomic disruption via CRISPR (Fig. 3F). Unlike wild-type cells, REDD1-deficient R28 cells did not exhibit an increase in DCF fluorescence upon exposure to hyperglycemic conditions (Fig. 3G, 3H). In agreement with this finding, REDD1 deletion also prevented hyperglycemia-induced ROS in MEF in culture (Fig. 3I). To further investigate the mechanism whereby REDD1 contributes to increased ROS levels, we evaluated mitochondrial superoxide in live cells. R28 cells exposed to hyperglycemic conditions exhibited an increase in mitochondrial superoxide, whereas the effect was prevented by REDD1 deletion (Fig. 3J). Due to the importance of ΔΨ_m_ in ROS production,^11^ we also assessed the impact of hyperglycemic conditions on mitochondrial membrane potential using JC-1 dye. In wild-type R28 cells, exposure to hyperglycemic conditions increased the red fluorescent signal derived from JC-1 aggregates, implying an increase in the mitochondrial membrane potential (Fig. 3K). In REDD1-deficient cells, the ratio of JC-1 aggregate/monomer was similar to that observed in wild-type cells cultured in medium containing 5 mM glucose and, unlike in wild-type cells, did not increase upon exposure to hyperglycemic conditions (Fig. 3L).

### NAC Prevents Glucose-Induced ROS, REDD1 Expression, and Cell Death in R28 Cells in Culture

**Figure 4:**
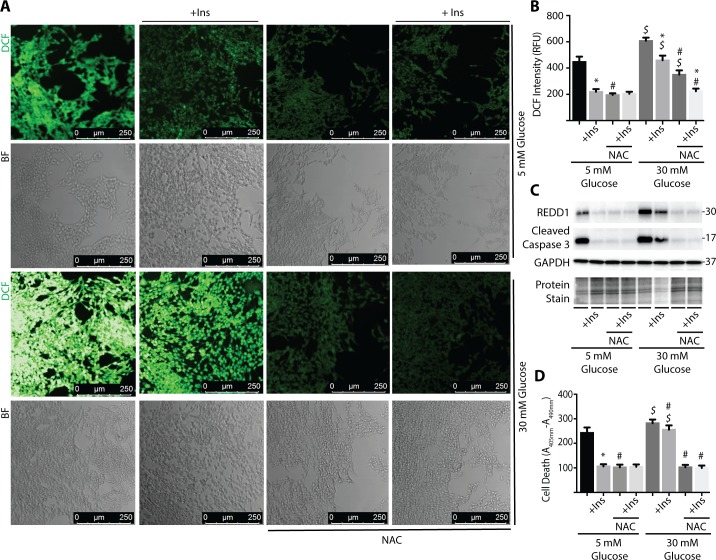
NAC prevents high glucose-induced ROS, REDD1 expression, and cell death in R28 cells. R28 cells were cultured in serum-free medium containing 5 or 30 mmol/L glucose plus the presence or absence of 100 nmol/L insulin (+Ins) and/or 10 mmol/L NAC for 24 hours. DCFDA was used to visualize ROS by confocal laser microscopy (A) and quantified using a plate reader (B). (C) Expression of REDD1, caspase-3 cleavage, and GAPDH were assessed via Western blotting. Gel loading was evaluated by protein stain. Protein molecular mass (kDa) is indicated at right of blots. (D) Relative cell death was assessed by ELISA for cytoplasmic nucleosomes. Results are representative for three experiments. Within each experiment, 9 to 10 independent samples were analyzed. Values are means + SEM. Statistically significant differences (P < 0.05) are denoted by * for Veh versus insulin, # for Veh versus NAC, and $ for 5 mM versus 30 mM glucose.

In addition to hyperglycemic culture conditions, REDD1 expression is also induced by serum deprivation.^35^ We recently demonstrated that insulin addition to serum-deficient culture medium prevented an increase in REDD1 protein expression in R28 cells.^22^ To further evaluate the relationship between ROS, REDD1 expression, and cell death, R28 cells were exposed to serum-deficient culture medium containing 5 or 30 mM glucose, as well as the presence or absence of insulin and/or NAC. In the present study, insulin and/or NAC addition to serum-deficient culture medium containing 5 mM glucose reduced ROS (Fig 4A, 4B), REDD1 expression (Fig. 4C), and cell death as assessed by caspase-3 cleavage (Fig. 4C) or DNA fragmentation (Fig. 4D). Similar to the effects observed with complete culture medium (Fig. 3), cells exposed to serum-deficient medium containing 30 mM glucose exhibited an increase in ROS, REDD1 expression, and cell death as compared to cells exposed to serum-deficient medium containing 5 mM glucose. While the effect of hyperglycemic conditions was still present, the magnitude of effect was notably reduced in the absence of serum (i.e., comparing Fig. 3 versus Fig. 4). Addition of either insulin or NAC to serum-deficient culture medium containing 30 mM glucose reduced DCF fluorescence, REDD1 expression, and cell death. Thus, ROS scavenging by NAC was sufficient to repress both REDD1 expression and cell death in response to serum deprivation or hyperglycemic conditions.

### ROS Promotes REDD1 Expression to Attenuate Akt/GSK3 Phosphorylation in R28 Cells in Culture

**Figure 5:**
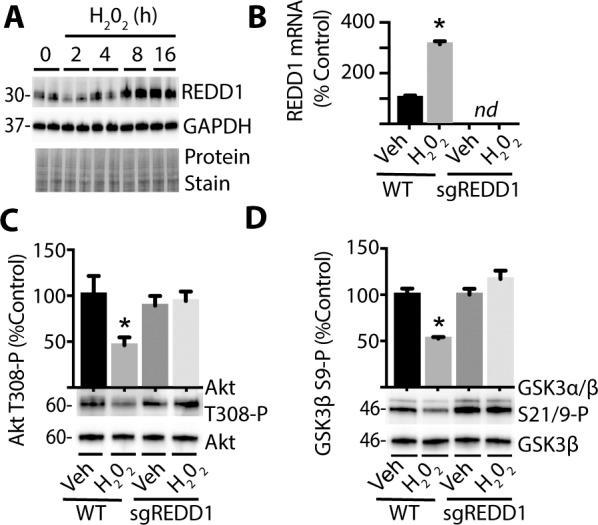
ROS promotes REDD1-dependent inhibition of Akt/GSK3 phosphorylation. R28 WT and REDD1 KO (sgREDD1) cells were exposed to culture medium containing 1 mmol/L H_2_O_2_ for 0 to 24 hours. (A) REDD1 and GAPDH protein expression were assessed via Western blotting. Protein loading was evaluated by protein stain. Protein molecular mass (kDa) is indicated at left of blots. (B) REDD1 mRNA was evaluated by qPCR 24 hours after addition of either H_2_O_2_ or Veh to culture medium. Akt phosphorylation at Thr308 (C) and GSK3α/β phosphorylation at Ser21/9 (D) were assessed via Western blotting 24 hours after addition of H_2_O_2_ or Veh. Results are representative for three experiments. Within each experiment, two to three independent samples were analyzed. Values are means + SEM. *P < 0.05 versus Veh; nd, not detected.

Because increased ROS levels were necessary for hyperglycemia-induced REDD1, we wanted to determine if enhanced ROS levels were sufficient to promote REDD1 expression. In wild-type R28 cells, addition of the exogenous ROS source H_2_O_2_ to culture medium increased the expression of REDD1 protein (Fig. 5A) and mRNA (Fig. 5B) expression. In addition, H_2_O_2_ attenuated phosphorylation of Akt at Thr308 (Fig. 5C) and GSK3β at Ser9 (Fig. 5D). In contrast, cells deficient in REDD1 did not exhibit attenuated phosphorylation of Akt or GSK3β upon exposure to H_2_O_2_.

### REDD1 Activates a Multicomponent Feedback Loop to Promote ROS Levels

**Figure 6:**
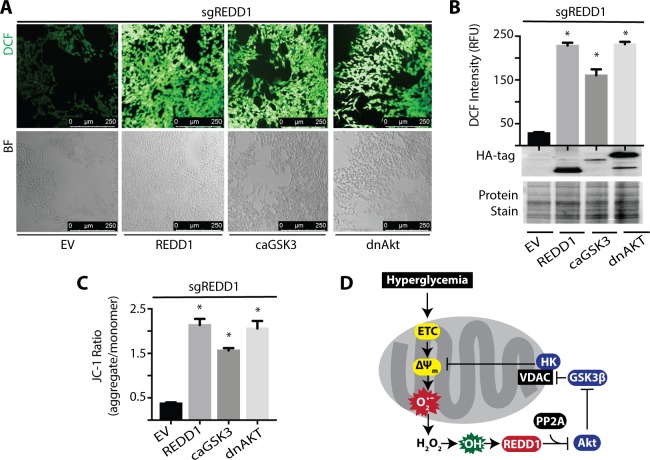
Constitutively active GSK3 or dominant negative AKT are sufficient to increase ROS and enhance the mitochondrial membrane potential in REDD1 KO cells exposed to hyperglycemic conditions. (A–C) REDD1 KO (sgREDD1) R28 cells were maintained in DMEM containing 5 mmol/L glucose supplemented with 10% FBS. Cells were transfected with either an empty vector (EV) control plasmid or constructs that express Hemagglutinin (HA)-tagged REDD1, HA-tagged constitutively active GSK3^S9A^ (caGSK3), or HA-tagged dominant negative Akt^K147M^ (dnAkt). Cells were then exposed to culture medium containing 30 mmol/L glucose for 24 hours. DCFDA was used to visualize ROS by confocal microscopy (A) and quantified (B). (B) HA-tagged protein expression was assessed via Western blotting. Gel loading was evaluated by protein stain. (C) Mitochondrial membrane potential was evaluated with JC-1 dye. The ratio of JC-1 aggregates to monomers were quantified. Results are representative for three experiments. Within each experiment, seven independent samples were analyzed. Values are means + SEM. *, P < 0.05 versus EV. (D) Working model for the mechanism whereby REDD1 expression promotes a ROS-generating feedback loop in response to hyperglycemia. ns, nonspecific.

We next investigated the mechanism responsible for the reduction in hyperglycemia-induced ROS upon REDD1 deletion. In REDD1-deficient R28 cells exposed to hyperglycemic conditions, restoring REDD1 expression was sufficient to promote ROS levels (Fig. 6A, 6B). Similarly, expression of either constitutively active GSK3^S9A^ (caGSK3) or dominant negative Akt^K147M^ (dnAkt) promoted ROS levels in REDD1-deficient cells as compared to an empty vector. REDD1, caGSK3, or dnAkt expression were also sufficient to increase the mitochondrial membrane potential in REDD1-deficient R28 cells exposed to hyperglycemic conditions (Fig. 6C). Overall, these findings support a model wherein REDD1 activates a ROS-generating feedback loop in response to hyperglycemic conditions (Fig. 6D).

## Discussion

The current study investigated the role of REDD1- and diabetes-induced oxidative stress in retinal apoptosis and visual dysfunction. In a recent study, we demonstrated that REDD1 protein expression is increased in the retina of diabetic mice, and deletion of REDD1 is sufficient to prevent diabetes-induced retinal cell death, electroretinogram (ERG) defects, and impaired visual thresholds.^22^ The protective effect of REDD1 deletion on diabetes-induced deficits is consistent with functional expression of REDD1 in the inner retina. Herein, we provide new insight into the mechanism responsible for the protective effect of REDD1 deletion in the retina of diabetic mice. Overall, the findings support a model whereby diabetes-induced hyperglycemia promotes REDD1 expression in a manner that activates a ROS-generating feedback loop, leading to increased retinal cell death and visual dysfunction (Fig. 6D).

In the present study, the functional consequence of diabetes-induced oxidative stress in the retina was evaluated by treating mice with the antioxidant NAC. NAC is a precursor of the amino acid cysteine, which is required for synthesis of the tripeptide glutathione (GSH). Reduced GSH is the most abundant antioxidant in human cells and plays a critical role in cellular redox signaling.^36^ In diabetic patients, GSH synthesis is diminished due to limited cysteine availability.^37^ In fact, cysteine and glycine supplementation to restore GSH synthesis normalizes elevated plasma markers of oxidative stress and lipid peroxides in diabetic patients.^37^ In this study, NAC supplementation normalized ROS levels in the retina of diabetic mice. In addition, NAC reduced REDD1 protein expression and markers of cell death in the retina of diabetic mice. NAC supplementation also improved visual function in diabetic mice as assessed by measurement of the optomotor response. We recently reported that REDD1 deletion prevents the development of visual deficits in STZ-diabetic mice.^22^ Whereas wild-type diabetic mice exhibit deficits in both visual acuity and contrast sensitivity, neither of these measures of functional vision are reduced in diabetic REDD1 knockout mice as compared to nondiabetic REDD1 knockout mice.^22^ Herein, NAC supplementation prevented increased REDD1 expression in the retina of STZ-diabetic mice. Similar to the principal effect of REDD1 deletion in the previous study,^22^ NAC supplementation also protected contrast sensitivity.

While it is well-established that DR is associated with decline in contrast sensitivity,^38^,^39^ diabetic patients without clinical signs of retinopathy also exhibit a reduction in contrast sensitivity.^40^,^41^ Contrast sensitivity is a particularly important element of visual function in low light conditions, when differences in luminance make distinguishing borders and details more difficult. Notably, contrast sensitivity is principally associated with inner retinal information processing, as compared to the outer retina and photoreceptor function. An important caveat is that the effects of NAC on the retina may not be direct but rather secondary to improved glucose homeostasis. Consistent with the previous report,^42^ STZ-diabetic mice receiving NAC in this study did not exhibit a significant reduction in blood glucose levels. Regardless, improved visual function in diabetic mice receiving NAC is not likely due exclusively to events restricted to the retina.

The findings here provide what we believe to be new evidence that REDD1 plays an important role in oxidative stress in the retina of diabetic mice. Specifically, REDD1 deletion prevented a diabetes-induced increase in retinal DCF fluorescence. ROS levels were similar in wild-type and REDD1-deficient R28 and MEF cells when cultured in medium containing 5 mM glucose. Wild-type cells exhibited increased REDD1 expression and enhanced ROS levels upon exposure to medium containing 30 mM glucose; however, REDD1-deficient cells did not. We previously demonstrated that hyperglycemia promotes REDD1 protein expression in the retina of diabetic mice.^21^ In diabetic mice treated with phloridzin to promote renal glucosuria and normalize blood glucose levels, retinal REDD1 expression was similar to that observed in nondiabetic controls. In the present study, ROS scavenging by NAC prevented increased REDD1 expression in the retina of diabetic mice and in cells in culture exposed to hyperglycemic conditions. Furthermore, increasing cellular ROS via an exogenous source (i.e., H_2_O_2_ addition to culture medium) was sufficient to enhance REDD1 protein expression. Thus, REDD1 is not the only important regulator of cellular ROS in response to hyperglycemic conditions; ROS also regulate REDD1 expression.

Ellisen et al.^18^ first demonstrated that REDD1 promotes ROS levels in TP63-null fibroblasts. In that same year, Shoshani et al.^17^ found that REDD1 expression was dramatically upregulated in ischemic cells of neuronal origin and promoted cell death in neuron-like PC12 cells by increasing sensitivity to oxidative stress. Consistent with the findings here, cells lacking REDD1 cultured in medium containing 25 mM glucose exhibit lower ROS levels as compared to wild-type cells.^19^ Reduced ROS in REDD1-deficient cells is linked to an effect on mitochondria and is due, at least in part, to enhanced antioxidant activity of thioredoxin.^19^ Thioredoxin is a major regulator of cellular redox signaling and plays a critical role in protecting cells from oxidative stress.^43^ Qiao et al.^19^ demonstrated that REDD1 forms a stress-induced complex with thioredoxin-interacting protein (TXNIP). TXNIP inhibits thioredoxin antioxidant function by binding to its redox-active cysteine residues.^44^ In both REDD1^−/−^ and TXNIP^−/−^ cells, thioredoxin activity is elevated and ROS levels are reduced.^19^ In REDD1^−/−^ cells, increased TXNIP expression was insufficient to increase ROS levels.^19^ With regard to DR, TXNIP expression is upregulated in the retina of diabetic mice, and TXNIP knockdown is sufficient to reduce diabetes-induced retinal pathology.^45^

In addition to binding to TXNIP,^19^ REDD1 also coimmunoprecipitates with PP2A and Akt.^16^ Retinal Akt kinase activity is attenuated as early as 4 weeks after the onset of diabetes^46^ and is associated with an increase in REDD1 protein expression.^22^ In fact, REDD1 deletion prevents the suppressive effect of diabetes on Akt activity in retinal lysates.^22^ In R28 retinal cells exposed to hyperglycemic conditions, enhanced REDD1 protein expression mediates a reduction in Akt phosphorylation.^22^ Enhanced REDD1 expression is also associated with attenuated phosphorylation of GSK3.^16^ In the present study, H_2_O_2_ addition to culture medium failed to suppress Akt or GSK3β phosphorylation in REDD1-deficient cells. Overall, the findings here are consistent with a model wherein hyperglycemia-induced REDD1 expression inhibits Akt and activates GSK3. In retinal cells exposed to hyperglycemic condition, REDD1 protein expression, mitochondrial membrane potential, and ROS levels were increased. REDD1 deletion prevented the increase in mitochondrial membrane potential and normalized ROS levels upon exposure to hyperglycemic conditions. Moreover, expression of a dominant negative variant of Akt or a constitutively active variant of GSK3 increased the mitochondrial membrane potential and ROS levels in the absence of REDD1. This supports a model proposed by Giacco et al.^8^ wherein hyperglycemic conditions activate a multicomponent feedback loop involving PP2A, Akt, and GSK3 to maintain a high mitochondrial membrane potential and promote mitochondrial ROS production. Thus, REDD1 expression reduces the antioxidant capacity of cells by promoting TXNIP-dependent inhibition of thioredoxin and enhances mitochondrial ROS production via PP2A-dependent repression of Akt.

The findings here support the conclusion that hyperglycemia-induced ROS promote REDD1 expression to activate a ROS-generating feedback loop that causes diabetes-induced retinal defects. DR is a highly prevalent diabetic complication that is clinically defined as a disease of the retinal microvasculature. However, loss of neurovascular coupling, neurodegeneration, gliosis, and neuroinflammation manifest before clinically visible vascular pathologies.^41^,^47^–^49^ In fact, recent advances in multifocal ERG demonstrate that neuroretinal defects precede and even predict the development of DR.^48^,^50^ Thus, there is an urgent need for interventions that prevent the onset of disease or arrest its progression prior to a clinical diagnosis that is based on overt vascular defects. It is important that therapeutic approaches targeting REDD1 expression and ROS in retina may represent novel interventions to address the early molecular events that lead to visual deficits in diabetic patients.
